# Elucidation of the Ro-Vibrational Band Structures in the Silicon Tetrafluoride Spectra from Accurate Ab Initio Calculations

**DOI:** 10.3390/molecules30214239

**Published:** 2025-10-30

**Authors:** Oleg Egorov, Michaël Rey

**Affiliations:** 1 Laboratory of Theoretical Spectroscopy, V.E. Zuev Institute of Atmospheric Optics SB RAS 1, Akademician Zuev Sq., Tomsk 634055, Russia; oleg.egorov@iao.ru; 2 Groupe de Spectrométrie Moléculaire et Atmosphérique UMR CNRS 7331, UFR Sciences BP 1039, 51687 Reims, France

**Keywords:** SiF_4_, silicon tetrafluoride, ab initio calculations, global line list, effective model, infrared spectra

## Abstract

We report the construction of comprehensive line lists for the three stable isotopologues of silicon tetrafluoride (^28^SiF_4_, ^29^SiF_4_, and ^30^SiF_4_) using a new effective Hamiltonian and dipole moment model built from accurate ab initio potential energy and dipole moment surfaces developed in this work. The vibrational energy levels were grouped into a series of polyads up to *P_max_* = 19, while the ro-vibrational energy levels were computed up to *J_max_* = 99. Each line list covers the spectral range 0–2500 cm^−1^ and contains almost 500 million transitions at *T* = 296 K, with each being generated from 685 vibrational states and sub-states. Most of the cold and hot band transitions computed in this work were not available in the literature beforehand. The absorption cross-sections computed from the produced line lists were successfully validated by direct comparison with the experimental data measured by Pacific Northwest National Laboratory at room temperature. Most of the ro-vibrational band structures observed in the experimental spectra can now be elucidated using the line lists proposed in this work.

## 1. Introduction

Chemically, silicon tetrafluoride (SiF_4_) is a gas with high thermal stability compared to other silicon tetrahalides. The strong chemical bonds in SiF_4_ can be explained by its orbital energies: the 2p valence orbitals of each F atom closely match the energy of the 3s and 3p valence orbitals of the silicon atom. As a result, the Si–F bond length in SiF_4_ is unusually short compared to that in SiHF_3_, SiH_2_F_2_, and SiH_3_F (see, e.g., Wang et al. [1] for further details).

Environmentally, SiF_4_ is a pollutant gas that is widely produced in microelectronics as a byproduct of the etching of Si-based materials (Hada et al. [2]). Since fluorine is monoisotopic, ^28^SiF_4_ holds potential for producing isotopically pure (^28^Si) semiconductors (Ernst et al. [3]).

The abundance of SiF_4_ is related to that of hydrogen fluoride (HF). The highly toxic HF can be produced by hydrolysis of SiF_4_, which is a complex multistage chemical process (Ignatov et al. [4], Sennikov et al. [5]). HF synthesis is also possible via a mixture of SiF_4_ and H_2_ under low-pressure microwave plasma conditions (Liu et al. [6]). SiF_4_ has been detected in volcanic plumes of different volcanoes, in particular Mt. Etna, Vulcano (by Francis et al. [7]), Popocatépetl (by Love et al. [8], Stremme et al. [9], Taquet et al. [10,11]), and Satsuma-Iwojima (by Mori et al. [12]). It is assumed that SiF_4_ arises from the interaction between magmatic HF and siliceous rocks (SiO_2_).

From a spectroscopic point of view, SiF_4_ is a heavy, rigid molecule with only small amplitude motions whose vibrations exhibit a harmonic character up to high vibrational quantum numbers. This enables fast convergence of the Taylor series expansions, making the use of empirically based effective models relevant (see, e.g., the empirical studies on overtone and combination bands by Patterson and Pine [13] and McDowell et al. [14] and the theoretical study by Wang et al. [15] on the high-order canonical Van Vleck perturbation theory).

Due to its tetrahedral (*T*_d_) symmetry, SiF_4_ has no permanent dipole moment, which makes its pure rotational transitions extremely weak and challenging to measure (see, e.g., the recent paper by Simon et al. [16] on CF_4_). Early spectroscopic studies first focused on the infrared active fundamental bands ν_3_(*F*_2_) and ν_4_(*F*_2_) of the main ^28^SiF_4_ isotopologue. High-resolution Doppler-limited analyses of ν_3_(*F*_2_) and ν_4_(*F*_2_) were conducted by Patterson et al. [17] and McDowell et al. [14], respectively, using tunable diode lasers. Takami and Kuze [18] measured rotational transitions within the excited (0010) vibrational state using an infrared-microwave double resonance technique, which allowed the determination of rotational and centrifugal distortion parameters for the ground vibrational state. Later, Jörissen et al. [19] significantly extended the set of empirically determined effective Hamiltonian parameters by combining new infrared data for the ν_3_(*F*_2_) band with rotational transitions, including those of the ground vibrational state (see also reference [20]).

Unlike early works, the recent high-resolution studies by Boudon et al. [21,22] and Merkulova et al. [23] were focused on all three isotopologues simultaneously: ^28^SiF_4_, ^29^SiF_4_, and ^30^SiF_4_. Given the relatively high abundances of ^29^SiF_4_ and ^30^SiF_4_ (4.7% and 3.1%, respectively), some of their absorption features are clearly distinguishable in the spectrum of natural SiF_4_ (hereafter, “natural” means that all the three isotopologues are presented), particularly in the region of the strongest ν_3_(*F*_2_) band. Boudon et al. [21] used FTIR measurements based on the synchrotron radiation to assign the ν_3_(*F*_2_) and ν_4_(*F*_2_) bands of all three isotopologues. Moreover, the line positions of the 2ν_4_(*F*_2_) band of ^28^SiF_4_ were assigned for the first time. The line positions of the combination bands ν_2_ + ν_3_(*F*_1_, *F*_2_), ν_1_ + ν_4_(*F*_2_), and ν_2_ + ν_4_(*F*_1_, *F*_2_) were also assigned and fitted for ^28^SiF_4_, while ν_1_ + ν_3_(*F*_2_) was analyzed for all three isotopologues in reference [23].

Despite recent progress, the available room temperature line lists for such heavy molecules are far from complete. Indeed, at *T* = 296 K, SiF_4_ exhibits a very dense and congested spectrum due to having a tremendous number of hot transitions. As a result, most of the recent studies have been essentially focused on lower temperatures (e.g., at *T* = 160 K in reference [21]). As demonstrated in this work, nearly half a billion transitions were needed to converge the sum of intensities at *T* = 296 K in the spectral region of 0–2500 cm^−1^, using a new effective model which is able to predict more than 2000 bands and sub-bands. Needless to say, such a vast amount of data is difficult to extract from experimental spectra. Therefore, this work extends the current knowledge on the SiF_4_ spectra and supplements the results presented in the recently made empirical database TFSiCaSDa (Richard et al. [24]).

In this study, we present global line lists for ^28^SiF_4_, ^29^SiF_4_, and ^30^SiF_4_ computed from extensive quantum-chemical and advanced nuclear-motion calculations. First, ab initio potential energy and dipole moment surfaces (hereafter, PES and DMS) have been constructed and presented in Section 2 and Section 3. Then, the Watson–Eckart nuclear-motion Hamiltonian—was employed to compute variational eigenpairs up to *J* = 15 before a numerical block-diagonalization procedure was applied to build a full effective polyad model (Section 4). The ro-vibrational energy levels enabled computation of the partition function required for the Boltzmann distribution (Section 5). We also investigated how optimal cut-off values for the rotational angular momentum (*J*) and line intensities can be chosen (Section 6). Finally, simulated absorption cross-sections of natural SiF_4_ were validated against experimental data from the Pacific Northwest National Laboratory (hereafter, PNNL) [25] (Section 7).

## 2. Analytical Model of the PES and DMS

The analytical model of the PES (V) and DMS (μ) was expressed as a Taylor series expansion in terms of irreducible tensor operators (ITOs) $\Omega_{j}^{p}$:

(1) $$V\;\mathrm{or}\;\mu =\sum_{p=0}^{p_{\max}}\sum_{j}C_{j}\cdot \Omega_{j}^{p},$$

where $C_{j}$ represents expansion coefficients to be fitted, while each ITO of degree *p* in the symmetry coordinates is expressed as follows:

(2) $$\Omega_{j}^{p}=\sum_{i=1}^{n}T_{i}\cdot {\left[S_{1}^{A_{1}}\right]}^{p_{i}^{1}}\cdot {\left[S_{2}^{E}\right]}^{p_{i}^{2}}\cdot {\left[S_{3}^{F_{2}}\right]}^{p_{i}^{3}}\cdot {\left[S_{4}^{F_{2}}\right]}^{p_{i}^{4}}.$$

**Figure 1:**
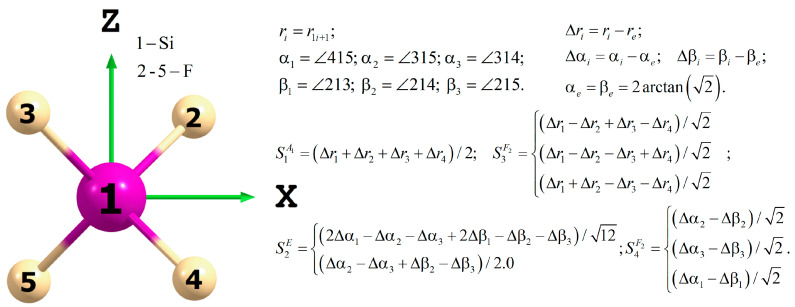
Definition of the internal and symmetry coordinates for SiF_4_.

Here, the expansion coefficients $T_{i}$ are computed from the *T*_d_ Clebsch–Gordan coupling coefficients. Each term in Equation (1) is of order *p* and transforms as the totally irreducible representation $A_{1}$ of the *T*_d_ point group for the PES and as $F_{2}$ for the DMS. Since SiF_4_ is a spherical top molecule, only one component of the DMS is necessary. In this work, we considered the x component ($\mu \equiv \mu_{x}$). The definition of the one—($S_{1}^{A_{1}}$), two—($S_{2}^{E}$), and three—($S_{3}^{F_{2}}$ and $S_{4}^{F_{2}}$) dimensional symmetry coordinates as a function of the internal ones is given in Figure 1.

The use of symmetry coordinates combined with ITOs allows us to significantly reduce the number of unknown expansion coefficients $C_{j}$, as each ITO in Equation (2) is a polynomial function in *S*. As a result, a compact set of linearly independent parameters are obtained from the fit to the ab initio grid, even for non-Abelian point groups such as *T*_d_. Previously, the ITO formalism was successfully employed to construct the PESs of different polyatomic molecules, including those with *T*_d_ symmetry such as CF_4_ [26].

The grid of reference nuclear configurations was made by varying the symmetry coordinates according to the nonzero symmetry components of ITOs. As illustrated in Figure 1, the deviation of the symmetry coordinates from zero results in corresponding deviations of the internal coordinates from their equilibrium values. However, to ensure an unambiguous correspondence between the internal coordinates used in this work and those employed in ab initio packages such as MOLPRO, all ab initio calculations were performed using the 3*N* Cartesian coordinates (here, *N* = 5). The Cartesian coordinates were obtained by solving a system of 3*N* equations:

(3) $$\left\{\begin{array}{l} S=I,\\ \sum_{i=1}^{5}m_{i}d_{i}=0,\\ \sum_{i=1}^{5}m_{i}\left(a_{i}^{ref}\times d_{i}\right)=0. \end{array}\right..$$

In Equation (3), S is the column vector consisting of the nine symmetry coordinates (see Figure 1), while I represents their deviations from zero, which are unique for each point of our grid. The vectors $d_{i}$ denote the Cartesian displacements of the *i*-th atom from the reference Cartesian position contained in the vector $a_{i}^{ref}$. The last six equations in Equation (3) correspond to the first and second Eckart constraint conditions for rigid molecules (see, e.g., Papoušek and Aliev [27]). The reference Cartesian vector is defined such that the atoms are positioned at the corners of the cube, which has side lengths of $2r_{e}/\sqrt{3}$. The relation between the symmetry and Cartesian coordinates in Equation (3) was established by using the standard formulas:

(4) $$\left\{\begin{array}{l} q_{i}=a_{i+1}^{ref}+d_{i+1}-a_{1}^{ref}-d_{1},\\ r_{i}=\sqrt{q_{ix}^{2}+q_{iy}^{2}+q_{iz}^{2}},\;i=1..4;\\ \alpha_{i}\left(\beta_{i}\right)=\arccos\left[\left(q_{jx}q_{kx}+q_{jy}q_{ky}+q_{jz}q_{kz}\right)/\left(r_{j}r_{k}\right)\right],\;i=1..3,\\ j=3,2,2\left(1,1,1\right);k=4,4,3\left(2,3,4\right). \end{array}\right..$$

Finally, to fit the ab initio points, it is more convenient to use Morse-cosine functions

(5) $$\begin{array}{l} y_{1..4}=1-\exp\left[-1.5\left(r_{1..4}-r_{e}\right)\right],\;\\ a_{1..3}=\cos\left(\alpha_{1..3}\right)-\cos\left(\alpha_{e}\right),\;b_{1..3}=\cos\left(\beta_{1..3}\right)-\cos\left(\beta_{e}\right) \end{array}.$$

instead of the $\Delta r_{1..4}$, $\Delta \alpha_{1..3}$, and $\Delta \beta_{1..3}$ displacements involved in the symmetry coordinates (see Figure 1). The use of Equation (5) improves the asymptotic behavior of the model beyond the reference nuclear configurations.

## 3. Ab Initio Calculations

### 3.1. PES

The ab initio electronic structure calculation for SiF_4_ is computationally demanding because it requires computing the correlation energy for 50 electrons. To our knowledge, no full-dimensional ab initio PES for SiF_4_ has been developed so far. On the other hand, SiF_4_ is ideally suited to applying a single-reference approach, as the vertical excitation energies to the nearest triplet and single excited electronic states are extremely high compared to the IR spectral range of 98,700 and 100,250 cm^−1^, respectively, as estimated in this work at the CAS(24, 16)/MRCISD(Q)/AVQZ level of the theory.

To achieve an accuracy of ~1 cm^−1^ when predicting the vibrational band origins from the ab initio PES, the orbital basis set size must approach the complete basis set (CBS) limit. Furthermore, corrections due to high-order electronic correlations, scalar relativistic effects, and diagonal Born–Oppenheimer corrections (DBOCs) must also be included. In this study, we have developed two ab initio PESs for SiF_4_ (hereafter referred to as PES_I and PES_II), based on the simplified explicitly correlated [RHF-CCSD(T)-F12x{x = a, b}] methods by Knizia et al. [28] and implemented in MOLPRO (by Werner et al. [29,30]).

For PES_I, the RHF-CCSD(T)-F12a method was combined with the VTZ-F12 basis set. This type of approach is already used in the literature for Van der Waals complexes and other many-electron systems. The calculations were performed on a grid (Grid_I) consisting of 8440 nuclear configurations that were generated using the eighth order ITOs. The resulting ab initio energies were thus fitted using 192 expansion coefficients (see Equation (1)) with a root mean square (RMS) deviation of 0.02 cm^−1^.

According to Table 1, PES_I is able to predict the fundamental band origins with an average absolute error of ~4 cm^−1^. This turns out to be a good result given the relatively modest computational cost of the RHF-CCSD(T)-F12a/VTZ-F12 approach. Specifically, this approach is about 75 times faster per point than the “standard” RHF-CCSD(T) method combined with the aug-cc-pV5Z basis set, while it is only 7 times slower than the rough RHF-CCSD(T)/VTZ approach.

In order to compute the correlation energy from all electrons of SiF_4_ (i.e., core–core, core–valence, and valence–valence contributions), the RHF-CCSD(T)-F12b method was combined with the CVQZ-F12 basis set. Additionally, a smaller grid (Grid_II) consisting of 178 nuclear configurations was constructed using ITOs expanded up to the fourth order. Single-point calculations took up to 2 h for the *D*_2_ and *C*_2_ symmetries and up to 8 h without any symmetry.

Based on earlier benchmark calculations (e.g., for the S_2_O molecule with 40 electrons [31]), it is known that the explicitly correlated RHF-CCSD(T)-F12b/CVQZ-F12 approach provides results comparable with the aug-cc-pCV5Z basis set associated with the RHF-CCSD(T) method. However, when core electrons are included, corrections from high-order Slater determinants are required; otherwise, the predicted band origins tend to be overestimated. To address this, the CCSDT(Q)/VDZ approach was utilized in the MRCC package (by Kállay et al. [32,33]) for Grid_II. These calculations took from 3 to 9 h per point, depending on the molecular symmetry.

Next, we have computed the two energy differences: [RHF-CCSD(T)-F12b/CVQZ-F12]–[RHF-CCSD(T)-F12a/VTZ-F12] and [CCSDT(Q)–CCSD(T)]/VDZ. They were summed and then fitted using expansion (1) up to the fourth order, yielding a RMS deviation of 0.03 cm^−1^. The fitted parameters were used to predict the summed energy difference for all points of Grid_I, bringing them to the RHF-CCSD(T)-F12b/CVQZ-F12 + CCSDT(Q)/VDZ level of the theory. The fit of the corrected energies of Grid_I yielded PES_II, with the same number of expansion coefficients and the same RMS error as PES_I.

The fundamental band origins predicted using PES_II agree with the empirical values within 1 cm^−1^, with a RMS error of only 0.65 cm^−1^. To our knowledge, this represents the most accurate ab initio description to date of the fundamentals for a “heavy” *T*_d_-type molecule. For comparison, previous ab initio calculations for CF_4_ [26] reproduced the ν_3_ band with a discrepancy of 5 cm^−1^, using a smaller VQZ basis set without any high-level corrections. Consequently, the present results validate the use of advanced ab initio methods for rigid, many-electron molecules such as SiF_4_.

We should stress the fact that no scalar relativistic and DBOC corrections were included here because their contributions to the band origins are about an order of magnitude smaller than those from the high-order electronic correlations. Including such corrections is justified only when the ab initio energies are close to the CBS limit. As shown in Table 2, PES_II systematically underestimates all the fundamental band origins due to the lack of convergence of the ab initio energies with respect to the orbital basis set size. This is typical of the RHF-CCSD(T)-F12b/CVQZ-F12 approach.

Our final PES was obtained by refining the quadratic force constants of PES_II as well as the equilibrium geometry using the empirical data of Boudon et al. [22]. After refinement, the fundamental band origins now agree within 10^−3^ cm^−1^ while the equilibrium geometry matches within 10^−4^ Ang. (see Table 1).

### 3.2. DMS

The ab initio values of the electric dipole moment were calculated via the finite difference method as the first derivative of the potential energy with respect to the electric field: Δ*V*/Δ*f*. The field (*f*) was applied along the *x* axis with two magnitudes: 0.0005 and −0.0005 a.u.

In order to check the convergence with respect to the orbital basis set size, two approaches were tested: RHF-CCSD(T)-F12a/AVTZ and RHF-CCSD(T)-F12b/AVQZ. As illustrated in Table 2, the first derivatives of the DMS with respect to the normal mode coordinates *q*_3_(*F*_2_) and *q*_4_(*F*_2_) show good convergence, with a difference between the two approaches of 0.0003 and 0.0004 Debye for $\left|\partial \mu /\partial q_{3}\right|$ and $\left|\partial \mu /\partial q_{4}\right|$, respectively. Accordingly, the less computationally demanding RHF-CCSD(T)-F12a/AVTZ approach was used to calculate the ab initio values of the electric dipole over the full grid of 2385 nuclear configurations. This grid covers the sixth-order ITO polynomials of symmetry *F*_2_. The final fit of the ab initio reference values achieved a RMS error of 10^−5^ Debye using 273 expansion parameters.

According to Table 2, the first derivatives of our ab initio DMS are in an excellent agreement with the empirical results obtained by Burtsev et al. [34] from the Raman spectrum of crystalline SiF_4_, originally measured by Bernstein et al. [36]. It is worth mentioning that the values of the first derivatives obtained from an empirical effective model differ from those given in Table 2. According to the recent empirical study by Boudon et al. [21], the effective first derivative extracted from the analysis of the line intensities of the ν_3_(*F*_2_) band was estimated as 0.5444 (38) Debye. This is about 28% larger than the present result. We suggest two main reasons to explain such a discrepancy. First, the missing hot transitions may have a significant impact on the spectrum of the gaseous SiF_4_, even at lower temperatures (in particular at *T* = 160 K, as considered in reference [21]), making the extraction of the line intensities of the cold bands hazardous without support of theoretical predictions. Second, only a limited number of effective dipole moment parameters was determined from analysis of the high-resolution spectra. Finally, most of the resonance interactions between the energy levels were omitted in the empirical models, which led to an incomplete set of diagonal and non-diagonal parameters. Here, the term “diagonal” refers to a parameter associated with creation and annihilation operators with the same powers. Undoubtedly, the spectral analysis using a global effective model with ab initio-determined parameters should provide more consistent results.

## 4. Effective Hamiltonian and Dipole Moment Operator

Variationally computing the energy levels and eigenfunctions of a five-atomic, heavy molecule like SiF_4_ remains a challenging task. In the previous study, the nuclear-motion problem was solved for CF_4_ [26] up to *J* = 80. In this work, higher *J* values are required to properly converge the integrated intensities. Within that context, a non-empirical effective model can be derived by applying a series of unitary transformations to the nuclear-motion Hamiltonian model, which is composed of a kinetic energy operator and the PES. As an alternative to Van Vleck, contact transformations based on perturbation theory, the novel methodology proposed in reference [37], were employed in this work. In this approach, instead of transforming the Hamiltonian, we search for a unitary transformation that brings selected variational eigenfunctions into a block-diagonal form, following a polyad scheme *P*. The corresponding matrix representation of the block-diagonal, effective Hamiltonian is thus obtained from the transformed eigenvectors and variational rotation-vibration energy levels. The key advantage of this approach is in the simultaneous construction of an effective dipole moment operator.

*Step 1: Variational calculation*. Nuclear-motion calculations were first performed using the “rigid” version of the computer code TENSOR [38], based on the Eckart–Watson [39] ro-vibrational Hamiltonian expressed in terms of normal-mode ITOs. Both the kinetic energy and potential parts were Taylor expanded at order 12 in terms of the nine coordinates (*q*_1_, *q*_2(*a*,*b*)_, *q*_3(*x*,*y*,*z*)_, *q*_4(*x*,*y*,*z*)_) describing the SiF_4_ vibrations, before the polynomial expansion was reduced at order 6, following the strategy of reference [38]. This strategy was already applied for computing accurate spectroscopic line lists of different semirigid molecules (for example, references [40,41,42]), including molecules with *T*_d_ symmetry [26]. For nonrigid molecules, the “hybrid” version of the TENSOR code [43] based on the Hougen–Bunker–Johns formalism was developed and successfully applied to the quasilinear triplet CH_2_ [44] as well as on the nonrigid NH_3_, CH_3_, and H_2_O_2_ molecules (see reference [43] and references therein).

To achieve a convergence better than 10^−3^ cm^−1^ for the vibrational levels up to 2500 cm^−1^, 15,708, 13,784, 29,454, 42,790, and 44,714 basis functions were used in the variational calculation for the symmetry blocks *A*_1_, *A*_2_, *E*, *F*_1_, and *F*_2_, respectively. Among all these basis functions, respectively, 753, 520, 1259, 1700, and 1933 functions were selected to define reduced vibrational eigenfunctions for solving the *J* > 0 problem. Like for CF_4_ or SF_6_, the normal-mode representation is very well suited for SiF_4_, allowing a fast convergence of both the Hamiltonian expansion and variational calculation. It is worth mentioning that the atomic masses were employed here to approximately account for non-adiabatic effects. In this work, the rotation-vibration eigenpairs were obtained and stored up to *J* = 15, before applying a series of transformations. Unlike DVR-like calculations, all quantum numbers are provided in a quite straightforward manner.

*Step 2: Effective model*. The variationally computed eigenvector matrices characterized by *J* and the total symmetry *C* were then block diagonalized using a transformation **T**^(*J*,*C*)^, following a specific polyad scheme in order to include the most relevant resonance coupling terms. A proper characterization of all the resonance couplings is very challenging when using an empirical approach, mostly due to missing information on the so-called “dark states” that may lead to poorly defined spectroscopic parameters. This is not the case when using ab initio calculations, which account for almost all possible resonances. In this work, an effective model was defined by following the polyad scheme *P* = 6υ_1_ + 2υ_2_ + 8υ_3_ + 3υ_4_, up to *P_max_* = 19, to cover the range 0–2500 cm^−1^. Note that the polyad number *P* = 1 is missing using this definition so that our rotation-vibrational states will be labelled by *J*, *C*, *n*, and *P*, with *P* = 0, 2, 3, …, 19. Here, *n* is a ranking number sorting the energy levels in increasing order for a given (*J*, *C*, *P*) block. At this stage, the obtained block-diagonal Hamiltonian matrices **H**^(*J*,*C*,*P*)^ up to *J* = 15 and *P* = 19 are nothing but matrix representations of an effective Hamiltonian with a set of parameters determined through an iterative procedure (see reference [37]).

In this work, a global effective Hamiltonian was expanded in creation-annihilation operators up to order 14 in order to include the band 7ν_2_. The maximum rotational degree was 6—except for the ground vibrational state where it was 8—resulting in 32,990 Hamiltonian parameters up to *P* = 19. For line intensity calculations, **T**^(*J*,*C*)^ was used to transform the matrix of the dipole moment components of the laboratory-fixed frame. Finally, all *P_m_*–*P_n_* (*m* = 0, …, 19, *n* = 0, …, 12) transitions were computed using 58,594 ITOs of symmetry *F*_2_.

**Figure 2:**
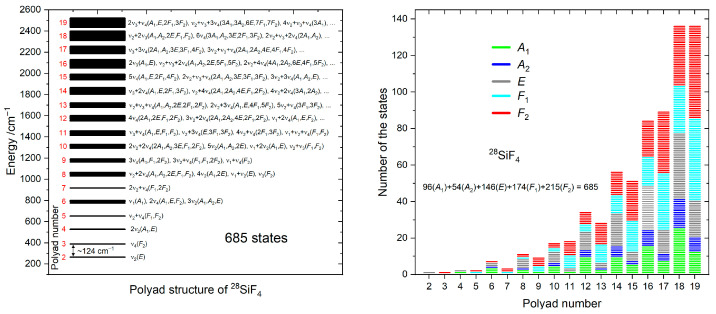
Polyad structure of the vibrational states of ^28^SiF_4_: energy scale (**left panel**); symmetries of the states inside each polyad (**right panel**).

The polyad structure of ^28^SiF_4_ obtained in this work is depicted in Figure 2 (left panel). A total of 685 excited vibrational sub-states were computed using this scheme. The energy gap between the lowest polyad is about 124 cm^−1^, but it decreases gradually for higher polyads, which include many more vibrational states. For example, each of the last two polyads, namely *P* = 18 and *P*= 19, contains 136 vibrational sub-states (Figure 2, right panel). A similar polyad scheme was applied for ^29^SiF_4_ and ^30^SiF_4_.

Finally, more than 95% of the vibrational band and sub-bands produced by our effective model have never been empirically studied so far. All these states led to a tremendous number of hot transitions required for converging the opacity at *T* = 296 K.

## 5. Partition Function

To estimate the maximum value *J_max_* for each polyad as well as the population of a ro-vibrational state at a given temperature (*T*), we can compute the partition function

(6) $$Q\left(T\right)=\sum_{j}g_{j}\exp\left(-\frac{h\cdot c\cdot E_{j}}{k_{B}T}\right),$$

where h [J × s] and $k_{B}$ [J × K^−1^] are the Planck and Boltzmann constants; c is the speed of light [cm × s^−1^]; $g_{j}$ is the statistical weight of the energy level $E_{j}$ [cm^−1^]. The total statistical weight is given by *g* = *g_ev_ g_rot_*, where *g_ev_* corresponds to the electronic and vibrational degeneracies, while the rotational part *g_rot_* is the product of the (2·*J* + 1) degeneracy in the absence of an external electromagnetic field with the nuclear spin statistical weight.

For the main isotopologue ^28^SiF_4_, the nuclear spins of the atoms are *I*(^28^Si) = 0 and *I*(^19^F) = 1/2. Thus, ^28^SiF_4_ has the same nuclear statistical weights as those of ^12^CH_4_, namely 5, 5, 2, 3, and 3 for the *A*_1_, *A*_2_, *E*, *F*_1_, and *F*_2_ symmetries of the *T*_d_ point group. ^30^SiF_4_ has the same weights since *I*(^30^Si) = 0. The case of the ^29^SiF_4_ isotopologue is different because of *I*(^29^Si) = 1/2 leading to a state-independent weight of 2. However, the state-independent weight does not change the line intensity and will therefore be omitted.

Equation (6) was summed over 4,826,347 energy levels computed from our new effective Hamiltonian. From the *direct* sum (6), the partition function of ^28^SiF_4_ is 442,170. Using the product approximation $Q=Q_{rot}Q_{vib}$, the value of the partition function differs by 0.5%, being 440,028. This approximation is used when a full set of energy levels is not available, in particular in the HITRAN database (see Gamache et al. [45]). For the two other isotopologues, the direct sum (6) gives 444,226 (^29^SiF_4_) and 446,257 (^30^SiF_4_), which are close, within 0.46% and 0.92%, to that of ^28^SiF_4_.

**Figure 3:**
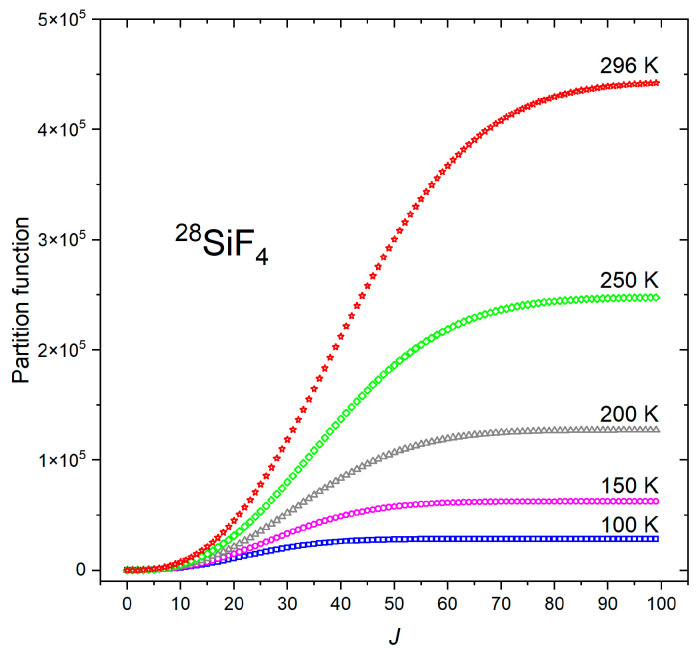
Convergence of the partition sum of ^28^SiF_4_ at different temperatures.

As shown in Figure 3, the partition function is converged for energy levels up to *J* = 60 for the temperature range *T* = 100–150 K. At room temperature (296 K), the difference between *J* = 98 and *J* = 99 is below 0.05%, so *J_max_* = 99 was considered in this study.

## 6. Cut-Off Values and Super-Lines

Selecting appropriate cut-off values for line intensities and the angular momentum *J* for each polyad of SiF_4_ can be a tricky task due to the huge number of the transitions. At a fixed intensity cut-off, the number of transitions may change significantly, even by incrementing *J*.

The intensities [cm^−1^/(molecule·cm^−2^)] of the ro-vibrational transitions were computed using the following formula

(7) $$I_{f\leftarrow i}=\frac{8\cdot \pi^{3}\cdot \nu_{if}}{3\cdot h\cdot c\cdot Q\left(T\right)}\cdot g_{i}\cdot \exp\left(-\frac{h\cdot c\cdot E_{i}}{k_{B}\cdot T}\right)\cdot \left(1-\exp\left(-\frac{h\cdot c\cdot \nu_{if}}{k_{B}\cdot T}\right)\right)\cdot S\left(f\leftarrow i\right),$$

where the subscripts *f* and *i* refer to the final (upper) and initial (lower) states; $\nu_{if}$ [cm^−1^] is the line position, $\nu_{if}=E_{f}-E_{i}$; other definitions are explained in Equation (6). The square of the matrix element of the molecular electric dipole moment known as the line strength (see, e.g., Bunker and Jensen [46]), S(f←i), was computed using the ab initio eigenfunctions of the effective Hamiltonian as well as the components of the dipole moment.

**Figure 4:**
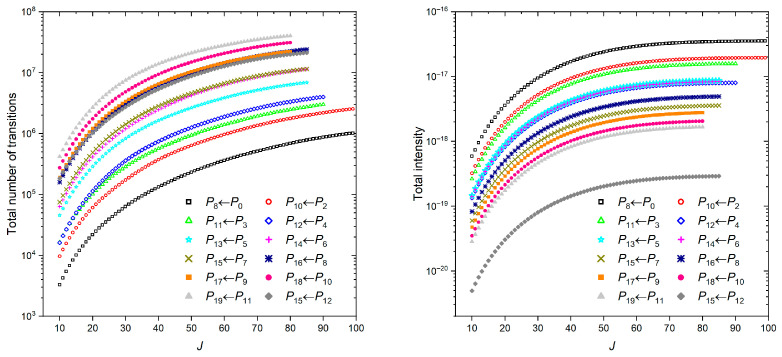
Number of the *P_m_*←*P_n_* transitions as a function of *J* (**left panel**). Convergence of the total intensity at *T* = 296 K for different *P_m_*←*P_n_* transitions (**right panel**).

The energy levels and transitions were computed up to *J_max_* = 99 for the first 10 polyads, namely *P* = 0 and *P* = 2–10. Above, the *J_max_* value was gradually decreased, namely from *J_max_* = 90 (*p* = 11, 12) and 85 (*P* = 13–16) to 80 (*P* = 17–19). The polyad transitions with the highest numbers of lines are displayed in Figure 4 (left panel). In terms of lines, the cold bands are dominated by *P*_8_←*P*_0_, which is composed of about ≈10^6^ lines, while the hot *P*_19_←*P*_11_ is composed of about ≈4 × 10^7^ lines. As indicated by the polyad structure given in Figure 2, both types of interpolyad transitions are characterized by Δυ_3_ = ±1, which corresponds to the strongest dipole derivative with respect to the normal coordinate *q*_3_ in SiF_4_ (see Table 2).

The total intensity of the transitions follows a Boltzmann distribution (Figure 4, right panel), meaning that the contribution of the hot transitions depends on the energy of the lower polyad. For that reason, the *P*_10_←*P*_2_ and *P*_11_←*P*_3_ hot transitions rank second and third, just after *P*_8_←*P*_0_. Interestingly, the combined absorption for these two hot transitions is comparable to that of the cold one, with intensities of 3.533 × 10^−17^ and 3.526 × 10^−17^ cm^−1^/(molecule·cm^−2^) (hereafter, cm/molecule), respectively.

Figure 4 also illustrates a typical difficulty when constructing line lists of “heavy” molecules: a large number of hot transitions (e.g., *P*_18_←*P*_10_ or *P*_19_←*P*_11_) composed mainly of weak lines is involved. Actually, such transitions form a quasi-continuum absorption background, which is not resolved because of the high density of lines but cannot be ignored for proper opacity calculations, at room temperature in particular.

Following the strategy that was previously established, in particular for CF_4_ [26] and SF_6_ [41], in order to make fast spectra simulations, we have defined so-called “strong” and “weak” lines. In this approach, it was suggested to model the quasi-continuum formed by the contributions of huge amounts of very weak lines using so-called “super-lines”, which represent integrated intensity contributions on a pre-defined grid of small wavenumber and temperature intervals. The initial line list for ^28^SiF_4_—computed using a cut-off of 10^−30^ cm/molecule for rotational transitions and of 10^−28^ cm/molecule otherwise—contained nearly half a billion transitions (448,594,361) over the range of 0–2500 cm^−1^. This list was subsequently reduced to 18,477,082 “strong” lines by applying a cutoff of 10^−25^ cm/molecule. The list composed by only “strong” lines covers the range 0–2100 cm^−1^, while the remaining transitions were converted to super-lines using a 10^−3^ cm^−1^ step size. The line list for ^29^SiF_4_ and ^30^SiF_4_ contains similar numbers for the transitions, as the isotopic abundance was fixed to 100% for all species.

## 7. Validation

### 7.1. General Comments

The experimental PNNL spectrum of SiF_4_ contains various impurities resulting from its hydrolysis. Among these, SiF_3_OSiF_3_ (hereafter, Si_2_F_6_O) is the most thermodynamically favorable product [4]. The presence of Si_2_F_6_O was noted in both early and recent experimental studies (see, e.g., references [14,22,23]). As will be demonstrated in this section, the contribution of Si_2_F_6_O in the PNNL cross-sections (measured at 298 K) is comparable in magnitude to some combination, overtone, and hot bands of SiF_4_.

The absorption cross-sections of the natural SiF_4_ were simulated using the line lists of all three isotopologues. To this end, the line intensities were scaled by the following natural abundances: 0.92223, 0.04685, and 0.03092 for ^28^SiF_4_, ^29^SiF_4_, and ^30^SiF_4_, respectively. A Lorentzian line profile with a constant half-width at half-maximum (HWHM) of 0.05 cm^−1^ was applied. The spectral step was set to 0.06 cm^−1^ and the simulation was conducted at a temperature of 296 K.

**Figure 5:**
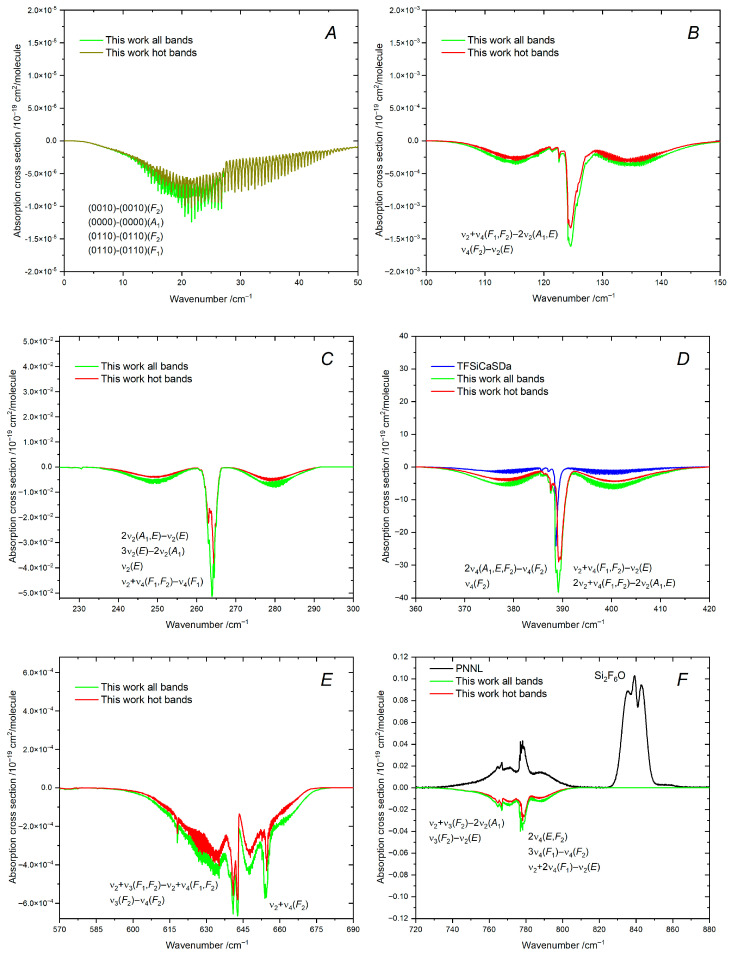

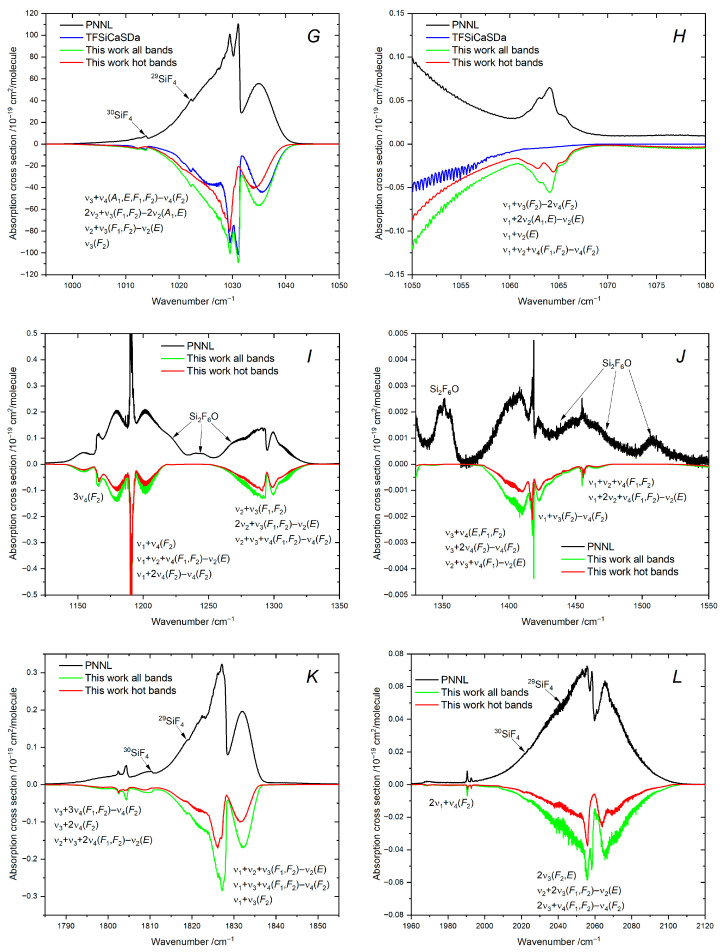
Absorption cross-sections of the natural silicon tetrafluoride (^28^SiF_4_, ^29^SiF_4_, and ^30^SiF_4_ isotopologues are included) simulated using line lists from this work in comparison with the observed data after 700 cm^−1^ (PNNL [25]). The strongest absorption features between 10 and 2120 cm^−1^ are displayed in Panels (**A**–**L**). The vibrational assignment of the dominant bands is given (their integrated intensities are presented in Table 3). The absorption due to Si_2_F_6_O is also displayed.

Table 3 gives the integrated intensities of the bands contributing significantly to the absorption, as displayed in Figure 5. As a comparison, the simulation based on the empirical TFSiCaSDa line list is also provided. According to reference [24], the TFSiCaSDa line list includes the transitions for the two fundamental bands, ν_4_(*F*_2_) and ν_3_(*F*_2_), as well as for several hot bands associated with Δυ_3_ = ±1. The line intensities for these hot bands were calculated using empirical dipole moment parameters derived from ν_3_(*F*_2_) and obtained in reference [21] from their analysis of individual line strengths. For the ν_4_(*F*_2_) band, the vibrational transition moment was estimated from the measured integrated intensity in reference [22].

The line lists developed in this work were based on a global ab initio approach accounting for almost all resonance interactions in a given polyad *P* (Figure 2). Moreover, a complete set of effective dipole moment parameters describing both cold and hot band transitions was derived from our ab initio DMS.

### 7.2. Region: 0–300 cm^−1^

Due to SiF_4_ being a spherical top molecule, its rotational spectrum is mainly induced by centrifugal distortion. As shown in Table 3 and Figure 5A, the rotational transitions within the excited (0010) state are the strongest (~6 × 10^−24^ cm/molecule), which is consistent with the high-resolution measurements by Takami and Kuze [18]. The rotational transitions within the ground vibrational states are much weaker [20].

The hot ν_4_(*F*_2_)–ν_2_(*E*) band located at 124 cm^−1^ also falls in this region (see Figure 5B). This hot band was clearly observed in the FTIR spectrum recorded at low temperature (160 K) by Boudon et al. [22], which led to a detailed analysis of its line positions. However, to our knowledge, the line intensities have not been empirically studied yet. As shown in Table 3, the integrated intensity of ν_4_(*F*_2_)–ν_2_(*E*) is about two orders of magnitude greater than that of the rotational bands.

The fundamental ν_2_(*E*) band located at 264 cm^−1^ (see Figure 5C) is not infrared active, unlike in the Raman spectrum of SiF_4_ (see, e.g., Clark and Rippon [47]). There is no first derivative of the electric DMS with respect to the normal mode coordinate *q*_2_(*E*). Consequently, the transition moment is governed by higher-order, rotational-dependent terms in the expansion of the effective dipole moment. Nevertheless, the absorption in this spectral region is stronger. The integrated intensity of ν_2_(*E*) is of the order of 10^−21^ cm/molecule, similar to that of the two hot bands: 2ν_2_(*E*)–ν_2_(*E*) and 2ν_2_(*A*_1_)–ν_2_(*E*) (see Table 3).

### 7.3. Region of ν_4_(F_2_)

The sum of intensities of the hot bands associated with Δυ_4_ = ±1 is larger than that of the fundamental ν_4_(*F*_2_) band located at 388 cm^−1^. As a result, the position of the total absorption peak is slightly shifted to the right from the origin of the ν_4_(*F*_2_) band. This is clearly seen when it is compared to the simulation based on the TFSiCaSDa line list where such hot bands are missing (see Figure 5D). The integrated intensity of ν_4_(*F*_2_) is 6% smaller in TFSiCaSDa compared to this work (see Table 3). The effective value of 0.3706 Debye for the first derivative of the DMS with respect to *q*_4_(*F*_2_) obtained in reference [22] without analysis of the individual line strength (i.e., under the zero-order approximation) is, on the contrary, 19% larger than the ab initio-based derivative obtained in this work (Table 2). This discrepancy may be explained by the zero-order approximation considered in reference [22].

In addition, there are other types of hot bands in the right wing of ν_4_(*F*_2_). Among these, we can mention the strongest one, namely ν_1_(*A*_1_)–ν_4_(*F*_2_), which is located at 412 cm^−1^. It was considered in reference [22] in order to help in the determination of the effective parameters of the fundamental ν_1_(*A*_1_) band. According to our line list, the integrated intensity of the ν_1_(*A*_1_)–ν_4_(*F*_2_) hot band is about two orders of magnitude smaller than that of the fundamental ν_4_(*F*_2_) band, which makes it not visible in Figure 5D.

### 7.4. Region: 570–880 cm^−1^

The hot bands with Δυ_3_ = Δυ_4_ = ±1 give the main contribution to the absorption in the spectral region at 643 cm^−1^ (see Figure 5E). This region also includes the combination band ν_2_ + ν_4_, located at 653 cm^−1^, that consists of the two sub-bands, namely ν_2_ + ν_4_(*F*_1_) and ν_2_ + ν_4_(*F*_2_), analyzed in reference [23]. However, the *F*_1_ sub-band is rather weak, while the integrated intensity of the *F*_2_ sub-band is one order of magnitude smaller than that of the ν_3_(*F*_2_)–ν_4_(*F*_2_) hot band (see Table 3).

The 2ν_4_(*E*) and 2ν_4_(*F*_2_) bands are responsible for the prominent absorption peak at 777 cm^−1^. Another peak at 767 cm^−1^ is mainly caused by the ν_3_(*F*_2_)–ν_2_(*E*) hot band. The line intensities of the fundamental ν_1_(*A*_1_) band at 800 cm^−1^ were too weak to include in the list of strong lines. For this reason, the ν_1_(*A*_1_) transitions were directly converted to super-lines. The strongest absorption peak presented in the PNNL, at 840 cm^−1^, originates from the antisymmetric Si–F stretching mode of Si_2_F_6_O. From Figure 5F, we can see that the maximum of the absorption due to Si_2_F_6_O is about two times larger than that of SiF_4_.

### 7.5. Region of ν_3_(F_2_)

Figure 5G displays the most famous spectral region of SiF_4_, corresponding to the strongest fundamental band ν_3_(*F*_2_). The integrated intensity of ν_3_(*F*_2_) presented in TFSiCaSDa is 11% larger than the present ab initio result (see Table 3). The results for ν_3_(*F*_2_) in TFSiCaSDa were probably biased by missing hot bands. According to reference [21], the effective value of the first derivative of the DMS with respect to *q*_3_(*F*_2_) was 0.5444(38) Debye. It is 28% larger than the ab initio value given in Table 2. Although the effective derivative cannot be directly compared with the ab initio one for different reasons (in particular, because the data in Table 2 correspond to the pure vibrational task, i.e., the contribution from the rotational operators is not included), we assume that the line intensities of ν_3_(*F*_2_) are slightly overestimated in TFSiCaSDa due to numerous hot transitions, which could not be accurately removed without support of ab initio predictions.

The integrated intensities of the hot bands presented in TFSiCaSDa, namely ν_3_ + ν_4_(*A*_1_, *E*, *F*_1_, *F*_2_)–ν_4_(*F*_2_) and ν_2_ + ν_3_(*F*_1_, *F*_2_)–ν_2_(*E*), differ from those in this work by 25% on average: the maximum difference is 53% [ν_3_ + ν_4_(*F*_2_)–ν_4_(*F*_2_)], while the minimum difference is 6% [ν_2_ + ν_3_(*F*_1_)–ν_2_(*E*)] (see Table 3). Interestingly, the total integrated intensity of those hot bands is only 8% larger than that obtained from the present line list. Nevertheless, the simulations based on the TFSiCaSDa line list underestimate the experiment, particularly in the left and right wings of ν_3_(*F*_2_) (see Figure 5G), mainly due to the absence of other hot bands that are relevant at *T* = 296 K.

### 7.6. Region: 1050–1550 cm^−1^

The first part of this region corresponds to the ν_1_ + ν_2_(*E*) combination band located around 1065 cm^−1^. Although the integrated intensity of ν_1_ + ν_2_(*E*) is of the order of 10^−20^ cm/molecule, this band is clearly seen in the PNNL spectrum (see Figure 5H). Note that the line positions of ν_1_ + ν_2_(*E*) have not been empirically studied so far because of their overlapping with the right wing of the strongest ν_3_(*F*_2_) band. There are hot bands with Δυ_1_ = Δυ_2_ = ±1 and the hot band ν_1_ + ν_3_(*F*_2_)–2ν_4_(*F*_2_) at 1051 cm^−1^; their integrated intensities are comparable (see Table 3).

Between 1167 and 1190 cm^−1^, there are two cold bands, namely 3ν_4_(*F*_2_) and ν_1_ + ν_4_(*F*_2_) (see Figure 5I). Quite surprisingly, the 3ν_4_(*F*_2_) band is stronger than the 2ν_4_(*F*_2_) band, while ν_1_ + ν_4_(*F*_2_) is the strongest combination band of SiF_4_. The second absorption peak around 1295 cm^−1^ originates from the *F*_1_ and *F*_2_ sub-bands of the combination band ν_2_ + ν_3_. The integrated intensity of the *F*_2_ sub-band is about 2.5 times larger than that of the first one.

The empirical line positions of ν_1_ + ν_4_(*F*_2_) and ν_2_ + ν_3_(*F*_1_, *F*_2_) were previously assigned in reference [23], based on spectra recorded at low temperature. However, these two bands alone are not sufficient to completely describe the absorption features observed in the room-temperature spectrum. Indeed, as seen in Table 3, the total absorption of the cold and hot bands becomes comparable at room temperature.

The absorption coefficient simulated using our line list shows a good agreement with the experimental measurements, except for several distinct absorption features (see Figure 5I). The unexplained feature observed between ν_1_ + ν_4_(*F*_2_) and ν_2_ + ν_3_(*F*_1_, *F*_2_) and located at 1243 cm^−1^ is likely caused by the antisymmetric Si-O stretching mode of Si_2_F_6_O. Other unexplained features clearly observed in the wings of the SiF_4_ bands are most likely due to the combination or overtone bands of Si_2_F_6_O. Indeed, this hypothesis comes from the fact that many of the fundamental frequencies of Si_2_F_6_O are below 1000 cm^−1^.

The two peaks in the last part of this spectral region correspond to ν_3_ + ν_4_ and ν_1_ + ν_2_ + ν_4_, at 1419 and 1455 cm^−1^, respectively. These two bands are rather weak and appear to be significantly perturbed in the PNNL spectrum (see Figure 5J). The remaining unexplained absorption features may be assigned to combination or overtone bands of Si_2_F_6_O, considering its previously established contribution (see, e.g., Figure 5F). The ν_3_ + ν_4_ band consists of the four sub-bands corresponding to the *A*_1_, *E*, *F*_1_, and *F*_2_ symmetries, while there are the *F*_1_ and *F*_2_ sub-bands in the ν_1_ + ν_2_ + ν_4_ band. In both cases, the strongest transitions belong to the *F*_2_ sub-bands, as expected. Currently, no high-resolution studies have been reported for these bands.

### 7.7. Region: 1790–2120 cm^−1^

The ν_1_ + ν_3_(*F*_2_) band at 1828 cm^−1^ is the second strongest combination band of SiF_4_, just after ν_1_ + ν_4_(*F*_2_). Some absorption features due to ^29^SiF_4_ and ^30^SiF_4_ are clearly visible in the left wing of ν_1_ + ν_3_(*F*_2_), similarly to the ν_3_(*F*_2_) band (see Figure 5K). The υ_3_(*F*_2_) mode has the largest isotopic shift among other fundamentals, respectively, of −9 and −17 cm^−1^ for ^29^SiF_4_ and ^30^SiF_4_. These pronounced shifts facilitate the study of the line parameters of the minor isotopologues, when υ_3_ is involved. For instance, the empirical line positions of the ν_1_ + ν_3_(*F*_2_) band were assigned in reference [23] for all three isotopologues. Note that ν_3_ + 2ν_4_ exhibits a noticeable absorption feature at the lower edge of ν_1_ + ν_3_(*F*_2_).

The two sub-bands 2ν_3_(*E*, *F*_2_) are responsible for the last prominent absorption peak of SiF_4_ at 2060 cm^−1^ (see Figure 5L). The absorption features due to ^29^SiF_4_ and ^30^SiF_4_ are less pronounced in the left wing of this band due to overlap with the nearby hot bands. As shown in Table 3, the integrated intensities of the hot bands are comparable to that of 2ν_3_, unlike in the region of the ν_1_ + ν_3_(*F*_2_) band.

The simulations based on the line list developed in this work show a good agreement with the PNNL in the regions of ν_1_ + ν_3_(*F*_2_) and 2ν_3_(*E*, *F*_2_). However, some absorption features are still absent in our simulations. The first one is the small peak located inside the ν_1_ + ν_3_(*F*_2_) band at 1822.5 cm^−1^. The simulated absorption is below the observation around 1837 cm^−1^. In the case of the 2ν_3_(*E*, *F*_2_) band, the experimental absorption profile looks stronger and broader near the band origin. All these unexplained features could also be attributed to Si_2_F_6_O because the polyad structure of our line list completely covers this region (see Figure 2).

## 8. Conclusions

This work clearly demonstrated the advantage of using ab initio calculations for the modelling of the room-temperature absorption spectrum of the silicon tetrafluoride molecule (SiF_4_). Both line positions and line intensities were computed using a new effective model that included all the cold bands and most of the hot bands within the spectral range of 0–2500 cm^−1^, where the most prominent absorption features of SiF_4_ are visible. To achieve this end, first full-dimensional potential energy and dipole moment surfaces were developed using high-level electronic structure calculations and including high-order electronic correlation effects [CCSDT(Q)]. For the first time, the fundamental band origins of ^28^SiF_4_ were predicted with an accuracy better than 1 cm^−1^, without any empirical corrections. The first derivatives of our ab initio DMS were found to be in excellent agreement with the empirical values derived from the Raman spectrum of crystalline SiF_4_ at 77 K.

The comprehensive line lists of ^28^SiF_4_, ^29^SiF_4_, and ^30^SiF_4_ constructed in this work were applied to analyze the room-temperature spectrum of the natural SiF_4_ measured by the Pacific Northwest National Laboratory (PNNL). Our analysis demonstrates that a large number of hot transitions are essential for achieving good agreement with observation. In many spectral regions—particularly those where ν_4_(*F*_2_) and ν_3_(*F*_2_) are involved—we have shown that the total integrated intensity of the hot transitions may exceed that of the cold bands. To significantly reduce the number of lines in our initial line lists containing almost half a billion transitions, all the weak transitions were converted into super-lines. A number of distinct features were also identified in the PNNL absorption spectrum and attributed to Si_2_F_6_O.

Although conventional effective empirical models can still be used to analyze low-temperature spectra, they strongly struggle to describe spectra at 296 K due to the absence of almost all of the hot band transitions. Finally, reliable ab initio predictions are crucial to accurately extracting the line intensities corresponding to both cold and hot bands. Only support from an ab initio model can provide such an amount of information.

The line lists developed in this work can be used for the simulation of the absorption spectrum of SiF_4_ at room temperature. Our results provide valuable spectroscopic support for future high-resolution studies, particularly those focused on line intensity analysis.

## Figures and Tables

**Table 1:** Fundamental band origins (in cm^−1^) of ^28^SiF_4_ variationally calculated (see Section 4) using the two “pure” ab initio PESs developed in this work.

Band	Empirical (Boudon et al. [22])	Ab Initio (This Work) ^1^		Final PES
		PES_I	PES_II	PES_II_Refined
ν_2_(*E*)	264.219525 (32)	261.462	263.582	264.2189
ν_4_(*F*_2_)	388.433276 (29)	384.898	387.785	388.4330
ν_1_(*A*_1_)	800.66566 (11)	795.882	799.989	800.6650
ν_3_(*F*_2_)	1031.544438 (65)	1025.575	1030.904	1031.5439
*r*_e_(Si–F)	1.5516985 (30)	1.558392	1.552765	1.551592

^1^ PES_I corresponds to the CCSD(T)-F12a/VTZ-F12 level of the theory; PES_II is based on the CCSD(T)-F12b/CVQZ-F12 method (core–core, core–valence, and valence–valence correlations are included) in combination with the correction from the high-order electronic correlations [CCSDT(Q)/VDZ]. The final PES was obtained from PES_II by the simultaneous empirical refining of its seconds derivatives (or harmonic frequencies) and equilibrium geometry (*r*_e_ in Ang.).

**Table 2:** Ab initio derivatives (in Debye, this work) with respect to the infrared modes *q*_3_ and *q*_4_ of ^28^SiF_4_.

Derivative	F12a/AVTZ ^1^	F12b/AVQZ	Empirical ^2^
$\left|\partial \mu /\partial q_{3}\right|$	0.4240	0.4243	0.42 ± 0.02
$\left|\partial \mu /\partial q_{4}\right|$	0.3112	0.3108	0.30 ± 0.01

^1^ The full notation of the ab initio method is RHF-CCSD(T)-F12x{x = a, b}. ^2^ Obtained by Burtsev et al. [34] following the approach of Haas and Hornig [35] and by using the experimental splitting of the transverse and longitudinal optical modes (TO and LO, respectively) measured by Bernstein et al. [36] from the Raman spectrum of crystalline SiF_4_ at 77 K.

**Table 3:** Integrated intensities of the strongest bands of ^28^SiF_4_ displayed in Figure 5.

Origin (cm^−1^)	Band	Region (cm^−1^)	*N*	*I*_ν_ (cm/molecule) ^1^	
				This Work	TFSiCaSDa
	(0010)–(0010)(*F*_2_)	1–57	58,547	6.363 × 10^−24^	
	(0000)–(0000)(*A*_1_)	5–28	9055	1.540 × 10^−24^	
	(0110)–(0110)(*F*_2_)	2–57	23,864	1.142 × 10^−24^	
	(0110)–(0110)(*F*_1_)	2–57	21,379	1.087 × 10^−24^	
124.175	ν_2_ + ν_4_(*F*_1_)–2ν_2_(*E*)	111–144	1444	2.015 × 10^−23^	
124.214	ν_4_(*F*_2_)–ν_2_(*E*)	106–147	11,554	2.650 × 10^−22^	
124.426	ν_2_ + ν_4_(*F*_2_)–2ν_2_(*E*)	109–139	1797	2.304 × 10^−23^	
125.907	ν_2_ + ν_4_(*F*_2_)–2ν_2_(*A*_1_)	111–145	3331	4.536 × 10^−23^	
263.204	2ν_2_(*A*_1_)–ν_2_(*E*)	240–286	4290	1.408 × 10^−21^	
263.669	3ν_2_(*E*)–2ν_2_(*A*_1_)	243–284	1590	4.030 × 10^−22^	
264.219	ν_2_(*E*)	236–290	9570	5.559 × 10^−21^	
264.646	ν_2_ + ν_4_(*F*_1_)–ν_4_(*F*_2_)	243–285	1739	4.123 × 10^−22^	
264.685	2ν_2_(*E*)–ν_2_(*E*)	239–289	3746	1.168 × 10^−21^	
264.897	ν_2_ + ν_4_(*F*_2_)–ν_4_(*F*_2_)	244–285	2193	5.396 × 10^−22^	
387.522	2ν_4_(*A*_1_)–ν_4_(*F*_2_)	359–418	99,620	6.361 × 10^−19^	
388.433	ν_4_(*F*_2_)	361–419	62,659	6.155 × 10^−18^	5.775 × 10^−18^
388.633	2ν_4_(*F*_2_)–ν_4_(*F*_2_)	361–420	163,332	1.894 × 10^−18^	
388.860	ν_2_ + ν_4_(*F*_1_)–ν_2_(*E*)	361–419	69,260	1.353 × 10^−18^	
389.025	2ν_4_(*E*)–ν_4_(*F*_2_)	361–422	97,931	1.132 × 10^−18^	
389.111	ν_2_ + ν_4_(*F*_2_)–ν_2_(*E*)	362–419	86,260	2.056 × 10^−18^	
389.536	2ν_2_ + ν_4_(*F*_1_)–2ν_2_(*E*)	362–420	97,970	4.352 × 10^−19^	
389.576	2ν_2_ + ν_4_(*F*_2_)–2ν_2_(*E*)	362–420	117,984	4.933 × 10^−19^	
391.057	2ν_2_ + ν_4_(*F*_2_)–2ν_2_(*A*_1_)	362–420	43,194	4.553 × 10^−19^	
640.597	ν_2_ + ν_3_(*F*_2_)–ν_2_ + ν_4_(*F*_2_)	610–663	2118	2.864 × 10^−23^	
640.848	ν_2_ + ν_3_(*F*_2_)–ν_2_ + ν_4_(*F*_1_)	608–665	2713	4.091 × 10^−23^	
642.119	ν_2_ + ν_3_(*F*_1_)–ν_2_ + ν_4_(*F*_2_)	612–665	2447	3.172 × 10^−23^	
642.370	ν_2_ + ν_3_(*F*_1_)–ν_2_ + ν_4_(*F*_1_)	610–663	1579	2.206 × 10^−23^	
643.111	ν_3_(*F*_2_)–ν_4_(*F*_2_)	602–672	18,227	4.499 × 10^−22^	
653.330	ν_2_ + ν_4_(*F*_2_)	639–672	2825	3.494 × 10^−23^	
766.504	ν_2_ + ν_3_(*F*_2_)–2ν_2_(*A*_1_)	741–798	2937	7.475 × 10^−22^	
767.325	ν_3_(*F*_2_)–ν_2_(*E*)	737–803	11,353	4.964 × 10^−21^	
777.066	2ν_4_(*F*_2_)	758–802	9678	5.829 × 10^−21^	
777.458	2ν_4_(*E*)	760–800	3184	1.339 × 10^−21^	
777.856	3ν_4_(*F*_1_)–ν_4_(*F*_2_)	761–798	3723	1.043 × 10^−21^	
778.296	ν_2_ + 2ν_4_(*F*_1_)–ν_2_(*E*)	763–799	3151	8.519 × 10^−22^	
1028.973	ν_3_ + ν_4_(*E*)–ν_4_(*F*_2_)	998–1062	58,012	2.899 × 10^−18^	2.477 × 10^−18^
1029.400	2ν_2_ + ν_3_(*F*_1_)–2ν_2_(*E*)	996–1060	113,020	2.301 × 10^−18^	
1029.708	ν_2_ + ν_3_(*F*_2_)–ν_2_(*E*)	991–1065	85,877	9.991 × 10^−18^	1.116 × 10^−17^
1030.174	ν_3_ + ν_4_(*F*_2_)–ν_4_(*F*_2_)	997–1060	63,366	3.541 × 10^−18^	5.420 × 10^−18^
1030.341	2ν_2_ + ν_3_(*F*_2_)–2ν_2_(*E*)	993–1063	211,923	3.007 × 10^−18^	
1030.445	ν_3_ + ν_4_(*F*_1_)–ν_4_(*F*_2_)	992–1064	121,231	6.275 × 10^−18^	7.309 × 10^−18^
1031.031	ν_3_ + ν_4_(*A*_1_)–ν_4_(*F*_2_)	1000–1062	63,240	3.031 × 10^−18^	1.624 × 10^−18^
1031.230	ν_2_ + ν_3_(*F*_1_)–ν_2_(*E*)	988–1068	88,859	9.425 × 10^−18^	9.985 × 10^−18^
1031.544	ν_3_(*F*_2_)	987–1068	53,957	3.491 × 10^−17^	3.860 × 10^−17^
1031.822	2ν_2_ + ν_3_(*F*_2_)–2ν_2_(*A*_1_)	995–1064	56,483	2.460 × 10^−18^	
1051.287	ν_1_ + ν_3_(*F*_2_)–2ν_4_(*F*_2_)	1014–1038	1226	2.464 × 10^−21^	
1063.382	ν_1_ + 2ν_2_(*A*_1_)–ν_2_(*E*)	1033–1083	7167	3.339 × 10^−21^	
1064.650	ν_1_ + ν_2_(*E*)	1034–1088	12,965	1.183 × 10^−20^	
1064.982	ν_1_ + 2ν_2_(*E*)–ν_2_(*E*)	1035–1086	8762	2.862 × 10^−21^	
1066.009	ν_1_ + ν_2_ + ν_4_(*F*_1_)–ν_4_(*F*_2_)	1039–1083	3588	1.002 × 10^−21^	
1066.292	ν_1_ + ν_2_ + ν_4_(*F*_2_)–ν_4_(*F*_2_)	1039–1083	4119	1.252 × 10^−21^	
1166.658	3ν_4_(*F*_2_)	1140–1192	13,478	1.793 × 10^−20^	
1190.005	ν_1_ + ν_4_(*F*_2_)	1161–1219	22,899	1.311 × 10^−19^	
1190.223	ν_1_ + ν_2_ + ν_4_(*F*_1_)–ν_2_(*E*)	1164–1217	18,203	3.133 × 10^−20^	
1190.506	ν_1_ + ν_2_ + ν_4_(*F*_2_)–ν_2_(*E*)	1164–1217	20,531	3.533 × 10^−20^	
1191.034	ν_1_ + 2ν_4_(*F*_2_)–ν_4_(*F*_2_)	1165–1217	28,004	3.722 × 10^−20^	
1293.927	ν_2_ + ν_3_(*F*_2_)	1248–1332	25,760	7.186 × 10^−20^	
1294.085	2ν_2_ + ν_3_(*F*_1_)–ν_2_(*E*)	1253–1326	24,701	2.108 × 10^−20^	
1294.790	ν_2_ + ν_3_ + ν_4_(*F*_2_)–ν_4_(*F*_2_)	1255–1326	20,682	1.084 × 10^−20^	
1294.892	ν_2_ + ν_3_ + ν_4_(*F*_1_)–ν_4_(*F*_2_)	1254–1326	18,596	1.070 × 10^−20^	
1295.026	2ν_2_ + ν_3_(*F*_2_)–ν_2_(*E*)	1251–1329	49,899	5.538 × 10^−20^	
1295.449	ν_2_ + ν_3_(*F*_1_)	1248–1329	20,422	2.746 × 10^−20^	
1417.406	ν_3_ + ν_4_(*E*)	1381–1439	7620	3.130 × 10^−22^	
1418.607	ν_3_ + ν_4_(*F*_2_)	1380–1441	9590	9.213 × 10^−22^	
1418.752	ν_3_ + 2ν_4_(*F*_2_)–ν_4_(*F*_2_)	1388–1448	14,347	2.304 × 10^−22^	
1418.878	ν_3_ + ν_4_(*F*_1_)	1392–1443	12,166	4.422 × 10^−22^	
1419.106	ν_2_ + ν_3_ + ν_4_(*F*_1_)–ν_2_(*E*)	1381–1439	10,396	3.187 × 10^−22^	
1439.920	ν_1_ + ν_3_(*F*_2_)–ν_4_(*F*_2_)	1415–1462	1888	2.241 × 10^−23^	
1454.442	ν_1_ + ν_2_ + ν_4_(*F*_1_)	1442–1471	488	5.751 × 10^−24^	
1454.725	ν_1_ + ν_2_ + ν_4_(*F*_2_)	1440–1472	3899	6.313 × 10^−23^	
1455.529	ν_1_ + 2ν_2_ + ν_4_(*F*_1_)–ν_2_(*E*)	1455–1470	219	2.253 × 10^−24^	
1455.577	ν_1_ + 2ν_2_ + ν_4_(*F*_2_)–ν_2_(*E*)	1444–1467	245	2.446 × 10^−24^	
1806.026	ν_3_ + 3ν_4_(*F*_1_)–ν_4_(*F*_2_)	1795–1836	2380	5.649 × 10^−22^	
1806.819	ν_3_ + 3ν_4_(*F*_2_)–ν_4_(*F*_2_)	1795–1836	1976	4.870 × 10^−22^	
1807.185	ν_3_ + 2ν_4_(*F*_2_)	1794–1837	8204	4.868 × 10^−21^	
1807.384	ν_2_ + ν_3_ + 2ν_4_(*F*_1_)–ν_2_(*E*)	1796–1837	2632	7.460 × 10^−22^	
1807.462	ν_2_ + ν_3_ + 2ν_4_(*F*_2_)–ν_2_(*E*)	1796–1835	3543	1.126 × 10^−21^	
1826.312	ν_1_ + ν_2_ + ν_3_(*F*_2_)–ν_2_(*E*)	1812–1836	11,704	2.802 × 10^−20^	
1827.820	ν_1_ + ν_2_ + ν_3_(*F*_1_)–ν_2_(*E*)	1812–1836	10,932	2.649 × 10^−20^	
1827.939	ν_1_ + ν_3_ + ν_4_(*F*_2_)–ν_4_(*F*_2_)	1814–1837	8106	9.321 × 10^−21^	
1828.179	ν_1_ + ν_3_ + ν_4_(*F*_1_)–ν_4_(*F*_2_)	1814–1837	14,154	1.621 × 10^−20^	
1828.353	ν_1_ + ν_3_(*F*_2_)	1814–1837	10,252	1.050 × 10^−19^	
1990.540	2ν_1_ + ν_4_(*F*_2_)	1975–2006	1641	3.350 × 10^−22^	
2056.109	ν_2_ + 2ν_3_(*F*_1_)–ν_2_(*E*)	2009–2098	16,402	1.632 × 10^−20^	
2057.601	ν_2_ + 2ν_3_(*F*_2_)–ν_2_(*E*)	2009–2098	14,291	1.378 × 10^−20^	
2059.017	2ν_3_(*F*_2_)	2007–2106	21,440	5.804 × 10^−20^	
2060.395	2ν_3_ + ν_4_(*F*_2_)–ν_4_(*F*_2_)	2008–2098	22,522	1.007 × 10^−20^	
2060.865	2ν_3_ + ν_4_(*F*_1_)–ν_4_(*F*_2_)	2008–2097	22,779	1.145 × 10^−20^	
2063.332	2ν_3_(*E*)	2008–2107	17,304	2.371 × 10^−20^	

^1^ The integrated intensities are the sum of the intensities of the ro-vibrational lines for a given band at *T* = 296 K. The isotopic abundance is not included. For further description of the TFSiCaSDa line list, see Richard et al. [24].

## Data Availability

The data that supports the findings of this study are available in the Supplementary Materials of this article.

## Supplementary Materials

The following supporting information can be downloaded at: https://www.mdpi.com/article/10.3390/molecules30214239/s1, the lists of super lines (“strong” and “weak” transitions) for silicon tetrafluoride (^28^SiF_4_, ^29^SiF_4_, and ^30^SiF_4_ isotopologues; isotopic abundancies are not included). The ab initio line lists with complete ro-vibrational assignment are available upon request. The ab initio values of the nuclear potential energy and electric dipole moment of SiF_4_ calculated in this work. The C++ and Fortran codes for computing potential energies from the final ab initio PES (“PES_II”).
